# Signals for Muscular Protein Turnover and Insulin Resistance in Critically Ill Patients: A Narrative Review

**DOI:** 10.3390/nu15051071

**Published:** 2023-02-21

**Authors:** Sebastián P. Chapela, Daniel Simancas-Racines, Martha Montalvan, Evelyn Frias-Toral, Alison Simancas-Racines, Giovanna Muscogiuri, Luigi Barrea, Gerardo Sarno, Pablo I. Martínez, María J. Reberendo, Natalia D. Llobera, Carlos A. Stella

**Affiliations:** 1 Departamento de Bioquímica, Facultad de Ciencias Médicas, Universidad de Buenos Aires, Ciudad Autónoma de Buenos Aires C1121ABE, Argentina; 2 Hospital Británico de Buenos Aires, Equipo de Soporte Nutricional, Ciudad Autónoma de Buenos Aires C1280AEB, Argentina; 3 Centro de Investigación en Salud Pública y Epidemiología Clínica (CISPEC), Facultad de Ciencias de la Salud Eugenio Espejo, Universidad UTE, Quito 170527, Ecuador; 4 Universidad Espíritu Santo, Samborondón 0901952, Ecuador; 5 School of Medicine, Universidad Católica Santiago de Guayaquil, Av. Pdte. Carlos Julio Arosemena Tola, Guayaquil 090615, Ecuador; 6 Carrera de Medicina Veterinaria, Facultad de Ciencias Agropecuarias y Recursos Naturales, Universidad Técnica de Cotopaxi, Latacunga 050108, Ecuador; 7 Investigador Asociado, Centro de Investigación en Salud Pública y Epidemiología Clínica (CISPEC), Facultad de Ciencias de la Salud Eugenio Espejo, Universidad UTE, Quito 170527, Ecuador; 8 Endocrinology Unit, Department of Clinical Medicine and Surgery, Federico II University Medical School of Naples, Via Sergio Pansini 5, 80131 Naples, Italy; 9 Centro Italiano per la Cura e IlBenessere del Paziente con Obesità (C.I.B.O), Department of Clinical Medicine and Surgery, Endocrinology Unit, Federico II University Medical School of Naples, Via Sergio Pansini 5, 80131 Naples, Italy; 10 Dipartimento di Scienze Umanistiche, Universit Telematica Pegaso, Via Porzio, Centro Isola F2, 80143 Naples, Italy; 11 Scuola Medica Salernitana, San Giovanni di Dio e RuggiD’Aragona University Hospital, 84131 Salerno, Italy; 12 Servicio de Terapia Intensiva, Hospital Británico de Buenos Aires, Ciudad Autónoma de Buenos Aires C1280AEB, Argentina

**Keywords:** muscle, critical illness, cytokines, proteolysis, sarcopenia

## Abstract

Sarcopenia in critically ill patients is a highly prevalent comorbidity. It is associated with a higher mortality rate, length of mechanical ventilation, and probability of being sent to a nursing home after the Intensive Care Unit (ICU). Despite the number of calories and proteins delivered, there is a complex network of signals of hormones and cytokines that affect muscle metabolism and its protein synthesis and breakdown in critically ill and chronic patients. To date, it is known that a higher number of proteins decreases mortality, but the exact amount needs to be clarified. This complex network of signals affects protein synthesis and breakdown. Some hormones regulate metabolism, such as insulin, insulin growth factor glucocorticoids, and growth hormone, whose secretion is affected by feeding states and inflammation. In addition, cytokines are involved, such as TNF-alpha and HIF-1. These hormones and cytokines have common pathways that activate muscle breakdown effectors, such as the ubiquitin–proteasome system, calpain, and caspase-3. These effectors are responsible for protein breakdown in muscles. Many trials have been conducted with hormones with different results but not with nutritional outcomes. This review examines the effect of hormones and cytokines on muscles. Knowing all the signals and pathways that affect protein synthesis and breakdown can be considered for future therapeutics.

## 1. Introduction

*Sarcopenia* is a syndrome in which the patient develops a continuous and generalized reduction in muscular mass and force along with physical limitations, deterioration of vital signs, poor quality of prognosis, and death [1]. This syndrome worsens the prognosis of all pathologies [2,3,4,5,6,7,8,9,10,11,12].

Patients with ICU-acquired weakness (ICUAW) and sarcopenic are no different. These sarcopenic individuals have increased morbidity, length of stay, duration of mechanical ventilation, and increased chance of being delivered to a retirement home [13,14,15,16,17].

Enormous efforts are being made to establish the amount and regimen of protein administration to acutely understrength patients. So far, it is understood that administration of a higher amount of protein is associated with a better prognosis [18,19,20]. However, a recent systematic review has shown conflicting evidence regarding protein intake. A meta-analysis showed that higher protein intake did not affect mortality, and small studies showed that it decreased muscle loss [21]. It is also known that underfeeding worsens the prognosis of individual patients [19]. It is not yet clear how much protein should be administered to patients with ICU-acquired weakness and sarcopenia. Guidelines from different societies have different recommendations on protein requirements: these specifications are expected to be between 1.2 and 2.0 g/kg of actual body weight daily [22], or, throughout the critical disease, 1.3 g/kg of protein per day can be given gradually [23].

Fasting triggers the release of counter-regulatory hormones, which enhance lipolysis and gluconeogenesis, with lactate, glycerol, and amino acids as substrates [24]. One of the origins of these amino acids is the breakdown of skeletal muscle [24]. However, fasting is not the only trigger for the release of counterregulatory hormones. Stress is also a trigger. Elevated cytokine levels over a prolonged period induce hormonal and metabolic changes in patients, leading to ICUAW and sarcopenia [25,26,27,28]. The same signals also trigger insulin resistance and hyperglycaemia in critically ill patients [29].

This article aims to review the nutritional, inflammatory, and endocrine signals that can affect muscle protein exchange and insulin resistance in sarcopenic patients.

## 2. Methodology 

Significant publications were considered for this narrative review. The search was done via PubMed using a combination of related search terms including “muscle”, “sarcopenia”, “critically ill”, “hormones”, “cytokines”, “insulin”, “glucocorticoids”, “growth hormone”, “adrenaline”, and “hypoxia”. According to their pertinency, the titles, and abstracts of the identified articles were examined by three members of the research team (S.P.Ch., E.F-T., and C.S.) and selected for full review if two authors agreed. Additionally, the references from the identified articles were also analysed in order to include these publications. Once all the articles were identified, the research team categorized each article based on the relationship between sarcopenia and the signals that lead to this muscle loss. Finally, the chosen papers underwent full content review for evidence of the association between sarcopenia and the extracellular or intracellular signals that lead to muscle loss.

## 3. Extracellular Signals Control Protein Turnover and Insulin Resistance in Muscle

**Figure 1:**
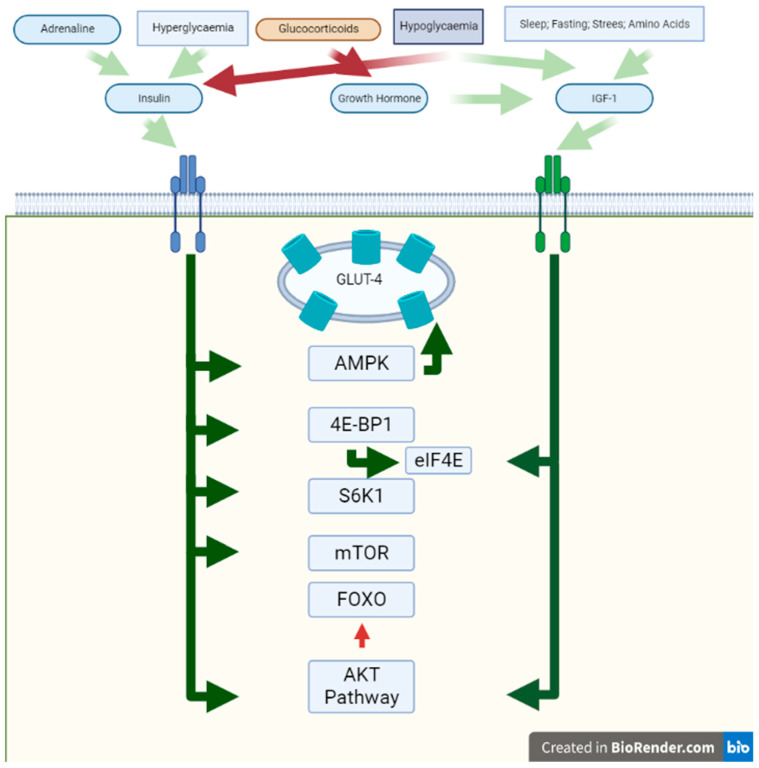
Anabolic effects on muscle. In this figure, the effects of activation of Insulin Receptor and IGF-R are shown. The white background represents the extracellular space, the green bar the sarcomere membrane, and the green background the cytosol. Red squares represent signals with an inhibitory effect and green squares represent stimulatory ones. The blue square represents hypoglycaemia, which has both inhibitory and stimulatory effects. Green arrows represent the stimulatory effect and red arrows represent the inhibitory effect on different proteins and pathways. Abbreviations: IGF-R: insulin-like growth factor receptor; AMPK = AMP-activated protein kinase.

**Figure 2:**
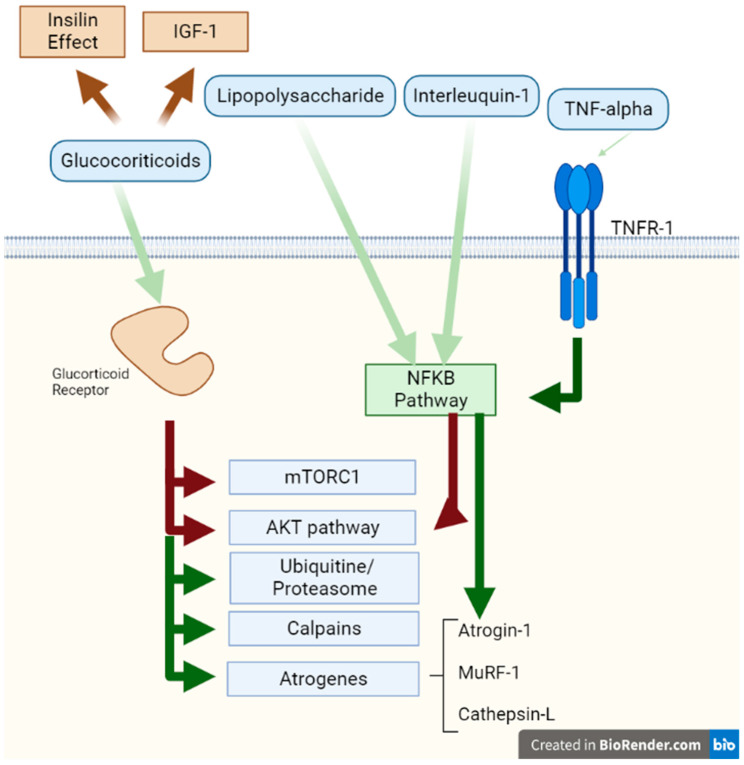
Catabolic effects on muscle. This figure represents the activation effects of the Glucocorticoid Receptor and the TNF receptor 1. The white background represents the extracellular space, the green bar the sarcomere membrane, and the green background the cytosol. Red arrows represent signals with an inhibitory effect and green arrows represent stimulatory effects on different proteins and pathways. Abbreviations: TNF: tumour necrosis factor; TNFR: tumour necrosis factor receptor.

The rate of protein synthesis in critically ill patients has a considerable variation [30]. This finding is not explained by the heterogeneity in the rate of protein synthesis but rather by the different types of proteins that these patients can synthesize [30]. The anabolic and catabolic effects of insulin and counter-regulatory hormones on muscles in critically ill patients has been known for many years. The pathophysiological effects of these hormones are known both from studies done in vitro and with patients. In addition, studies carried out in patients where an attempt was made to counteract the effect of these hormones in critical patients where metabolic changes related to these interventions were observed and described. None of these works had the objective of measuring muscle mass, muscle strength, or long-term functional outcomes, which are possible future research lines. In this section, we aim to describe the signalling that these effects produce as possible therapeutic targets, and highlight studies in which septic animals and patients with ICU-acquired weakness have been supplemented with these hormones. In addition, the stimuli for hormone secretion were analysed. Experimental trials in which patients were treated with hormones or with treatments that block the effect of hormones are reviewed. The summary of these effects is shown in Figure 1 and Figure 2.

### 3.1. Insulin

Insulin is an upregulated blood hormone that is released upon an elevation in blood glucose levels [31,32]. Insulin has a specific tyrosine kinase domain in the cell membrane [31,32]. The typically known effects of insulin are to increase glucose uptake by promoting translocation of GLUT transporters to the membrane, regulating critical enzymes of the glycolytic and gluconeogenic pathways, and regulating the expression of more than 100 specific genes [31,32]. More specifically, it stimulates glucose uptake at the muscle level and inhibits gluconeogenic precursor efflux into the liver [31,32]. Amino acid release from skeletal muscle during sepsis depends on a balance between protein synthesis and protein degradation rates, and insulin can modulate both metabolic processes under diverse physiological and pathological situations [31,32].

Insulin also promotes protein synthesis by accelerating mRNA translation, stimulating the translation mRNA binding step, and increasing the bioavailability of eIF4E through modulation by 4E-BP1 [33]. Insulin further mediates translation initiation by increasing the activity of S6K1, which phosphorylates the ribosomal target S6 [33]. The main actions of insulin on muscle proteins are summarized in Table 1.

On the other hand, insulin is responsible for the transport of glucose into the muscle. Glucose carrier 4 (GLUT4) is deposited in vesicles and readily translocates to the sarcolemma after the attachment of the insulin receptor or muscle contraction [34]. The insulin detection cascade implicates protein kinase B/AKT [34]. In addition, contraction-induced translocation of GLUT4 is regulated by phosphorylation of 59-adenosine monophosphate-activated protein kinase (AMPK) [34]. In addition, insulin induces contractility in cardiac muscle, as was shown in diabetic and nondiabetic subjects [35].

In physiologic conditions, insulin inhibits the FOXO pathway via AKT [36,37]. At the mRNA level, the essential insulin-dependent metabolic gene AKT2 and the down-regulated genes, namely FOXO3, were significantly upregulated in muscle parts with critical myopathy and without critical myopathy, compared to controls [34]. Insulin-dependent genes critical for the insulin signalling pathway upstream of AKT2, such as IGF1 receptor, IRS1, and PI3Kp110a, were markedly down-regulated in patients with or without acutely understrength myopathy compared to controls. In contrast, the levels of other insulin-dependent genes (INSR, IRS2, and SHIP2) did not differ between normal control subjects and acutely understrength patients [34]. Furthermore, metabolic acidosis is a metabolic disorder frequently found in critically ill patients [38,39]. This metabolic disorder leads to insulin resistance [40]. This finding has been reported in vitro in muscle myoblasts, where acidosis decreased insulin binding to its receptor, affecting the signalling cascade [40]. These findings, together with the high levels of counterregulatory hormones, are some of the possible causes that generate insulin resistance and its consequences in critically ill patients.

### 3.2. Growth Hormone

Growth hormone (GH) is synthesized in the hypophysis. Its supplementation to hormone-deficient patients reduced fat mass, increased lean biomass and muscle force, increased bone thickness, and increased the lipid profile [41]. Some stimulators for GH release are: drowsiness, fasting or hypoglycaemia, elevated insulin levels, stress, exercise, growth, and elevation of amino acids, such as arginine. Inhibitors of GH release are: hyperglycaemia, weight gain, glucocorticoid elevation, elderly age, and advanced pregnancy [41]. The effects of GH are primarily mediated by IGF-1 [42,43] and include: stimulation of lipolysis, increased protein synthesis and amino acid uptake, decreased liver glucose uptake, increased liver gluconeogenesis, and elevated insulin tolerance [41]. The main actions of GH on muscle proteins are described in Table 1.

In a randomized control trial (RCT), elective colonic surgery patients with total parenteral nutrition were randomized to receive GH (0.15 IU/kg/injection) or GH associated with IGF-1 (40 μg/kg/injection), or to a control group [38]. The GH and GH associated with IGF-1 groups had decreased urea levels; moreover, the GH–IGF-1 group exhibited conserved muscle protein synthesis. Finally, the GH group did not show changes in the branched-chain and aromatic amino acids groups; this could indicate an unaltered protein breakdown in muscles [44]. In addition, in another RCT, patients with severe sepsis treated with GH 0.1 mg/kg/day or placebo during the second, third, and fourth day after admission showed that GH administration decreased nitrogen production (*p* < 0.01) and nitrogen balance became positive in the GH group during treatment on day 3 (*p* < 0.05) [45].

Furthermore, in an RCT with acute sepsis patients treated with GH 0.1 mg/kg/day or placebo throughout four days after admission, the administration of GH decreased nitrogen production (*p* < 0.01), and the nitrogen balance became positive in the GH group during management on the third day (*p* < 0.05) [45]. In another study of GH in a population of burn patients, the administration of a daily dose of 0.2 mg/kg/day reduced wound healing times [43]. However, in an RCT of acutely understrength patients who received GH (daily dose, 0.10 ± 0.02 mg per kilogram of body weight) or placebo until discharge from intensive care or for up to 21 days, those receiving GH showed increased mortality (*p* < 0.001) [46].

### 3.3. Insulin-like Growth Factor 1 (IGF-1)

IGF-1 is an anabolic growth factor, synthesized mainly in the liver and stimulated by GH [47,48]. In muscle, this hormone attaches to the IGF receptor and triggers AKT, which integrates anabolic and catabolic feedback by disrupting substrates by phosphorylation [49]. One possible mechanism for IGF to increase protein synthesis is the alteration of eukaryotic initiation factor (eIF) activity through the phosphatidylinositol (PI) 3-kinase pathway involved in the phosphorylation of 4E-BP1 and p70. In addition, this hormone inhibits muscle protein degradation via activation of the AKT pathway, which phosphorylates and inhibits FOX O transcription factors [49]. As will be explained later, FOX O activates transcription of ubiquitin ligases such as muscle atrophy box 1 (MAXFb1) and muscle RING finger 1 (MuRF1), which are crucial for muscle protein degradation [49]. The main effects of IGF-1 on muscle proteins are described in Table 1.

In critically ill subjects, IGF-1 levels decrease between the day of admission and later in the ICU [48], but its relationship with death in critically ill subjects remains unclear. A study of 90 ICU patients showed no difference in IGF-1 levels in patients who died and the ones who survived in the ICU [48], but a study of 64 septic patients showed that septic subjects had lower IGF-1 values than normal volunteers, an inverse correlation with the SOFA score (Sequential Organ Failure Assessment), and patients who died in the ICU had lower IGF-1 levels [50]. In another study of 107 septic patients, IGF-1 and IGF-binding protein 3 (IGFBP3) were lower in septic patients compared to controls and they decreased with increasing severity of sepsis, but both levels were not associated with mortality [51]. In addition to its metabolic effects in muscles of septic rats, IGF-1 administration was found to improve memory of noxious arousal and spatial cognition and memories and inhibited apoptosis in the hippocampus [52]. However, in mechanically ventilated patients, IGF-1 levels were not related to dementia or the duration of normal brain state [53]. Similarly, in septic patients, an inverse correlation was observed between IGF-1 levels and intestinal bacterial translocation [54]. In an animal study model of sepsis, pretreatment with IGF-1 improved the bacterial clearance of *P. aeruginosa* in the liver and positively impacted survival [55].

Due to its known effect on muscle protein synthesis and because septic patients had lower levels of IGF-1, IGF-1 has become an interesting target to prevent muscle loss during sepsis. In rats, IGF-1 administration stimulates the phosphorylation of 4E-BP1, S6K1, and mTOR in non-septic and septic rat muscles [56]. Studies in patients with IGF-1 alone or in combination with IGFBP3 have been rather disappointing [57]. In an RCT of patients undergoing gastrectomy surgery, 80 µg/kg body weight was administered for five consecutive days, and a complete parenteral nutrition regimen standardized at 3 g/kg glucose and 0.1 g/kg nitrogen was administered [58]. Serum IGF-1 levels were higher, but IGF-1 did not improve nitrogen balance and the levels of 3-methylhistidine excretion did not show differences between the groups [58]. In another RCT, surgical trauma patients received GH alone or GH and IGF-1 or placebo; all patients received 28 kcal/kg/day and 0.15 g/kg/day of nitrogen [38]. GH–IGF-1 impeded the reduction in muscle polyribosomes, reflecting conservation of muscle protein synthesis, but no other changes in amino acid and glutathione metabolism were observed [44]. It is worth mentioning that the amount of nitrogen in both trials is not recommended today, so it will be interesting to evaluate the effect of this hormone in critically ill patients with current nutritional recommendations.

### 3.4. Glucocorticoids

In skeletal muscle, glucocorticoids (GCs) alter protein turnover, reduce protein synthesis rates, and enhance protein breakdown [59]. GCs act through a DNA-binding receptor. Their suppressive effect on protein synthesis comes from various mechanisms, including restriction of amino acid transport in muscles, which can limit protein synthesis and the stimulatory action of insulin, IGF-1, and amino acids (in particular leucine). However, the main restriction of protein synthesis is caused by the infringement of mTORC1, the kinase involved in phosphorylation of 4E-BP1 and S6K1 [59]. The predominant effects of GCs on muscle proteins are summarized in Table 1.

At the same time, several pieces of evidence support that GCs can mediate their catabolic actions by inhibiting the PI3K/AKT pathway, which mediates the anabolic actions of insulin/IGF-1 [59]. In addition, the stimulative effect of glucocorticoids on muscle proteolysis is the result of the mobilization of the ubiquitin–proteasome system, the lysosomal pathway, and the calcium-dependent mechanism (calpains) [60].

Protein degradation occurs mainly in myofibrillar proteins, muscle proteins, and extracellular matrix proteins [59]. GCs increase the expression of the FOXO transcription factor with the activation of various atrogenes such as Atrogin-1, MuRF-1, and Cathepsin-L [59,61]. GCs stimulate protein breakdown by stimulating the production of various components of the ubiquitin–proteasome system, either through the attachment of ubiquitin to the protein to be degrades or directly responsible for protein breakdown by the proteasome [59].

GCs impact the atrophy of different fibres, the most affected being fast-twitch or type II muscle fibres, while type I fibres are less impacted [59]. Furthermore, gravity and the catabolic mechanism of GCs may vary with age [59].

### 3.5. Glucagon

Glucagon is a catabolic hormone released by the pancreas, stimulating low glucose levels, nutrient or protein intake, and triggering the autonomic nervous systems [62]. The endocrine functions of glucagon oppose the effects of insulin [31]. During critical illness, glucagon levels are elevated; more specifically, active glucagon levels are increased [63]. This hormone stimulates liver glycogenolysis and gluconeogenesis, increasing glucose levels [63]. In addition, glucagon decreases serum amino acid levels [63].

Interestingly, in amino acid homeostasis, glucagon has receptors mainly in the liver, kidney, heart, adipose tissue, central nervous tract, adrenal gland, and spleen, but not in muscles [62]. The promotion of gluconeogenesis by this hormone requires the supply of glucose precursors such as lactate, alanine, and glycerol, but the increased supply of these compounds to the liver is not directly governed by glucagon [62].

In a post hoc analysis of the EPANIC study, critically ill patients showed glucagon concentrations at admission similar to controls, but increased from day 1 to day 7 [64]. In an animal model of sepsis, infusion of amino acids increased serum glucagon levels and catabolism of amino acids in the liver, while muscle wasting was not affected [64]. Neutralization of this hormone influenced glucose levels and lipid metabolism but not muscle wasting [64].

### 3.6. TNF Alpha

The inflammatory cytokine tumour necrosis factor (TNF-α) leads directly to damaged oxidative metabolism in skeletal muscle via signalling of the nuclear factor kappa B (NF-KB) inflammatory pathway [65,66]. TNF-α has two separate receptors: TNFR1 and TNFR2; in most cell types, NF-kB activation occurs primarily through TNFR1 [67].

This cytokine, through its receptor, activates the TAK1 protein and stimulates the I-KB kinase by inactivating I-KB, which is the natural repressor of NF-KB [67,68]. In this way, the transcription factor NF-KB is released and stimulates the expression of more than 150 genes [67,68].

However, NF-KB is not only triggered by TNF-α but by a wide diversity of different stimuli, including proinflammatory cytokines such as interleukin-1 (IL-1), T and B cell mitogens, bacteria and bacterial lipopolysaccharides (LPS), viruses, viral proteins, double-stranded RNA, and physical and chemical stresses [67]. The main effects of TNF-alpha on muscle proteins are summarized in Table 1.

Several pathways for the influence of TNF-α on protein catabolism in muscles have been described. Models of starvation-induced muscle atrophy demonstrate reduced function of the AKT pathway and up-regulation of the atrogin-1/MAFbx gene [69]. The mechanism involves the release of inhibition of FOXO1 and FOXO3 [70]. Elevated atrogin mRNA also correlates with increased TNF in inflammatory catabolic conditions [69].

In C2C12 cells, increased glucose uptake after exposing myotubes to TNF-α was accompanied by elevated levels of mRNA and protein of the glucose transporter molecule Glut-1 and increased abundance of mRNA transcripts and enzymatic activity of phosphofructokinase [71]. Furthermore, in C2C12 myotubes, TNF exposure increases atrogene mRNA and AKT-mediated canonical regulation of atrogenes is active [69].

In the same model, TNF does not affect Foxo1/3 mRNA levels or nuclear localization. In contrast, TNF increases nuclear Foxo4 protein; this finding implies that TNF individually increases AKT atrogene mRNA through FOXO4 [69]. Furthermore, in C2C12 cells, TNF-α stimulates UbcH2, a ubiquitin transporter protein whose upregulation is essential for enhancing ubiquitin-conjugating activity [66]. The UbcH2 promoter sequence contains a functional binding site for NF-KB; this binding to this region is enhanced upon TNF-α stimulation [66]. Finally, TNF-α in C2C12 myotubes increases the expression of FOXO1, FOXO3a, MAFbx1, and MuRF1 and decreases the expression of anabolic targets such as AKT, mTOR, P70S6k, and 4E-BP1 [72].

In COPD patients, TNF-α enhanced myotube glucose uptake and lactate output, as well as muscle glycolytic metabolism in an NF-KB-dependent manner, and triggered HIF (Hypoxia Inducible Factor) signalling [71].

### 3.7. Adrenaline

Adrenaline acts via B2 receptors and increases liver and muscular glycogenolysis. It also enhances hepatic gluconeogenesis. Finally, it increases the release of insulin and glucagon. Several years ago, studies showed that adrenaline has an anti-proteolytic effect on muscles [73,74]. A study of healthy volunteers infused with adrenaline, insulin, glucagon, and cortisol stimulated protein synthesis and breakdown [73].

A 2017 study in rats showed that adrenaline infusion suppresses the Ca2+-dependent pathway of protein breakdown and probably the calpain pathway [75]. In addition, there is evidence that adrenaline affects the ubiquitin–proteasome pathway [76]. This effect appears to be modulated by feeding status, with adrenal medulla resection and fed rats showing reduced muscle biomass and transverse area, whereas fasted rats increased proteolysis [73]. In isolated muscles, adrenaline suppressed the Ubiquitin–Proteasome pathway and the induction of atrogin-1 and MuRF1 after fasting [77].

### 3.8. Hypoxia-Inducible Factor

Hypoxia-inducible factors (HIFs) are transcription factors that are critical regulators of proper oxygen balance and are essential in growth, metabolism, and disease [78]. HIF-1α is activated by oxygen concentration [79]. HIF-1 stimulates glycolytic enzymes and the expression of genes that encode several types of glucose carriers [79]. HIF-1 is triggered in reaction to hypoxic events and promotes other genes and transcription signals to increase oxygen levels [78,79]. Such hypoxic states can be activated in numerous conditions like inflammation, sepsis, hypertension, hypovolemic shock, cardiac or pulmonary illness, and haemophilia [78,79]. 

In sepsis, HIF-1α interacts with pyruvate kinase M2, which inhibits pyruvate dehydrogenase, decreasing levels of acetyl-CoA and reducing coenzymes [80,81,82]. The Warburg effect allows coenzymes to be oxidized and reused in glycolysis, producing less ATP per glucose entering the cycle [80,81,82]. The main effects of HIF-1 on muscle proteins are summarized in Table 1.

Regarding hypoxemia in the ICU, information on prevalence is limited. In a multicentre prevalence study conducted in France, out of a group of 1604 patients, more than half had hypoxemia as identified by PAFI (PaO2/FiO2 ratio). ICU mortality was higher according to the level of hypoxemia, being higher in more hypoxemic patients, with moderate and severe hypoxemia also being identified as independent factors of death in the ICU [83].

In a study published in 2017, a group of British researchers (Caudwell Xtreme Everest Research Group) hypothesized that sarcopenia also occurs when exposed to high-altitude hypoxia. After analysing hypoxia-induced changes in body composition and identifying possible routes linked to loss of fat-free mass (FFM) and fat mass (FM), 24 researchers climbed from Kathmandu (1300 m) to the Everest base camp (EBC 5300 m) over 13 days [84]. It was observed that the higher altitude reached and the higher the level of hypoxemic hypoxia was accompanied by a gradual reduction in FM and FFM [84]. The changes in protein carbonyls were accompanied by a decrease in FM. On the other hand, 4-hydroxynonenal and IL-6 correlated with loss of FFM. Glucagon-like peptide-1 (GLP-1) (r = −0.45, *p* < 0.001) and the changes in nitrite concentration were related to the reduction of FFM [84].

In a multivariate model, GLP-1, insulin, and nitrite were predicted as significant factors for loss of FFM, while FM was predicted as significant in the loss of protein carbonyls. In another study published by Textoris et al. [85], the role of HIF1a as a prognostic biomarker in patients undergoing shock was evaluated. HIF1 controls the expression of genes associated with the cellular response to hypoxia [85,86,87].

HIF1a stimulates erythropoiesis, glycolysis, angiogenesis, and vasodilation [85,86,87]. In a study of 50 adult patients with shock and 11 healthy subjects, RNA was obtained from complete blood samples, and HIF1a expressed during the first 4 h of shock was assessed [85,86,87].

Higher HIF1a expression was observed in the patients with shock versus healthy individuals, and there was no significant difference in surviving versus non-surviving patients at 28 days [85]. Within the analysis of the work, HIF1a expression was not correlated with haemoglobin, PaO2, PaO2/FiO2 ratio, or mechanical ventilation [85].

Patients with pulmonary hypertension or persistent obstructive pulmonary disease (COPD) and chronic hypoxemia suffer from sarcopenia, which appears to be caused by a change in oxidative metabolism in muscle fibres (OXPHEN phenotype), inflammatory molecules leading to decreased protein synthesis and muscle atrophy (TNF α, IL-6, NF-Kb, m-TOR), imbalance of hormones related to the satiety mechanism (GLP-1/Leptin), or stimulation of oxygenation regulatory molecules (HIF α 1) [87,88]. In critically ill patients, inflammation is an installed process like hypoxemia in the previous case, and both plays in hypoxia and inflammation [89]. Oxygen detection mechanisms and hypoxia signalling are potential therapeutic objectives for treating acute and ongoing inflammatory diseases [90].

## 4. Signal Translation

To date, it has been shown how protein intake affects muscle protein synthesis and breakdown. Different hormonal signals affecting protein synthesis in the critical human patient were also reviewed as possible therapeutic tools. In the following sections, we will analyse the mechanisms of signal translation related to the hormones previously studied to understand the mechanisms involved in the translation of hormonal signals at different stages of the disease and possible therapeutic targets.

### 4.1. AMPK

AMP-activated protein kinase (AMPK) is an enzyme activated during changes in cellular metabolism, such as muscle contraction and hypoxia [91]. AMPK is an indispensable intermediary controlling vital cellular functions such as growth, proliferation, and surveillance [92]. In addition, it organizes multiple signalling pathways that control nutrient absorption and energy metabolism in different tissues. It provides a central underlying role in more complex physiological and behavioural phenomena, such as interorgan organization through various cytokines and adipokines, control of nutritional behaviour, voluntary energy expenditure, and cognitive capacity [92].

This protein kinase increases muscle wasting by promoting the expression of Atrogin1/MAFbx and MuRF1 genes [93]. AMPK activation also induces the C2 subunit of the proteasome, calpains, cathepsin B, and caspase-3 [94]. Finally, AMPK activation inhibits mTORC1, a kinase that regulates cell growth and will be described later [93]. In myotubes cultured with high CO_2_, AMPK was activated, leading to FOXO3a activation with increased MuRF1 and myotube atrophy [95].

### 4.2. AKT

AKT1 is a protein kinase that acts in several cellular mechanisms such as cell proliferation, migration, glucose metabolism, apoptosis, transcription, and others [96]. AKT1 induces protein synthesis pathways that lead to skeletal muscle hypertrophy and overall tissue outgrowth [97]. A mouse model using AKT1 showed its effects on growth and apoptosis in tissues other than muscle. Because it can inhibit apoptosis and improve cell viability, Akt1 has been involved as a crucial factor in many types of cancer [94]. Finally, AKT1 is involved in the PI3K/AKT/mTOR signalling pathway and other critical signalling mechanisms, such as FOXO [98].

### 4.3. GSK 3B

Glycogen synthase kinase 3 β, or GSK3B, is a kinase that phosphorylates and inactivates glycogen synthase [99,100]. Regarding glucose homeostasis, GSK3B negatively regulates several pathways leading to mitochondrial dysfunction and apoptotic pathways, among others [101,102,103,104,105,106]. GSK3β is a central kinase in inflammation, and the benefits of inhibiting this enzyme have been demonstrated in studies of sepsis and organ failure [101]. In a rat model of sepsis, the combination of selective GSK3β blockers reduced LPS-induced liver failure and injury [105]. GSK3β inhibitors reduced the severity of sepsis and improved survival in experiments in models of acute lung injury [101]. GSK3β kinase activity has also been implicated in organ damage due to hypoperfusion in septic shock [106,107,108].

GSK3β is a downstream effector of IGF-I/AKT signalling and is involved in the expression of atrogin-1 and MuRF1, and is induced by stimulation and atrophage [107]. In C2C12 cells with GSK-3β knockdown, corticosteroid administration suppressed atrogin-1 and MuRF1 expression, while pharmacological blockade of GSK-3β alone lowered atrogin-1 mRNA levels [107]. Further studies are needed to understand how GSK3β activity is dysregulated in the pathogenesis of sepsis and host immune responses to pathogens. It remains to be seen whether studies of small molecule inhibitors of GSK3β, that have produced striking data in animal models, can be translated to treating patients with this devastating disease.

### 4.4. mTOR

In mammals, the target of rapamycin (mTOR) is a kinase belonging to the phosphatidylinositol-related family of protein kinases 3. mTOR binds to various other proteins and provides the building block for two distinct protein complexes, the mTOR 1 complex and the mTOR 2 complex, which govern various cellular processes [109,110]. As a central member of the complex, mTOR operates as a protein kinase. This enzyme complex regulates pathways that control cell growth, survival, proliferation, movement, motility, protein synthesis, gene transcription, maintenance of the actin cytoskeleton, and autophagy [111,112]. mTOR also stimulates the activity of insulin receptors and insulin-like growth factor 1 receptors [113].

Activation of mTORC1 is required for myofibrillar muscle protein synthesis and skeletal muscle hypertrophy. This is induced in humans in response to physical exercise and ingestion of specific amino acids or amino acid derivatives [114]. Continued inactivation of mTORC1 signalling in skeletal muscle facilitates the loss of muscle mass and strength [110]. This occurs due to muscle wasting in advanced age, cancer cachexia, and muscle atrophy due to physical atrophy and inactivity [115].

### 4.5. Ribosomal Protein S6 Kinase 1 (P70S6k)

P70s6k induces the synthesis of cellular proteins [116,117]. Ribosomal protein S6 regulates mRNA translation through its localization at the tRNA attachment site on the 40S ribosome. Enhanced phosphorylation of ribosomal S6 upon activation of S6K1 corresponds to accelerated mRNA translation rates [33].

Phosphorylation of p70s6K is the hallmark of activation by mTOR [118]. There is uncertainty about the role of this activation in autophagy. For example, amino acids such as arginine and leucine can activate mTORC1 recruitment to lysosomes [118].

### 4.6. FOXO

FOXO is a group of transcription factors. FOXO3 induces ATROGIN-1 and causes atrophy of muscle fibres¿ [61] FOXO is inhibited by IGF-1 and AKT [61]. In addition, AMPK activates FOXO3a and induces the expression of autophagy-related proteins [93]. As seen above, CO_2_ increases AMPK levels, leading to activation of FOXO3a and MuRF1 expression [95]. In addition, TNF-α treatment of C2C12 myotubes increases FOXO transcription factor expression [72]. In human muscle, after endurance training, increased phosphorylation of AKT, GSK-3, and mTOR with decreased FOXO1 was observed; after detraining, decreased phosphorylation of AKT, GSK-3, and mTOR and increased FOXO1 was observed [119].

## 5. Effectors of Protein Breakdown

Previously, it was explained what is known about protein requirements and muscle, the signals in critically ill patients that affect muscles, and the translation of those signals. Finally, the effectors of those signals will be described.

### 5.1. Atrogin and MuRF1

The ubiquitin–proteasome complex is the major effector of the muscle protein breakdown pathway during muscle atrophy. Atrogin-1/MAFbx (atrogin) is a muscle-specific E3 protein involved in a chain reaction that is up-regulated in catabolic states, such as increased tumour necrosis factor exposure [69]. Atrogin-1 and MuRF-1 are two important muscle E3 ubiquitin ligases that are notably induced in many catabolic models in recognition of GC. Whereas FOXO upregulates Atrogin-1 gene transcription, MuRF-1 gene transcription is upregulated by FOXO (indirect transcriptional triggering) [59].

Overexpression of MAFbx in mouse myotubes resulted in atrophy, while deficiencies in MAFbx1 or MURF1 were resistant to atrophy [120]. The target substrates of MAFbx are suggested to be mainly regulatory proteins involved in muscle protein synthesis and regeneration, while MuRF-1 targets contractile and structural muscle proteins, but this is still under investigation [121,122].

Sepsis increases MAFbx and MuRF1 mRNA in the rat extensor digitorum and gastrocnemius muscles without elevation in the oxidative muscles [121]. IGF-1 therapy prevented the sepsis-induced increase in MAFbx mRNA and did not affect MuRF1. The sepsis-induced augmentation of MAFbx and MuRF1 in rodents depends on muscle fibre type, and their regulation occurs through unrelated routes [123].

In addition, as described above, GCs do activate UPP. Administering the synthetic glucocorticoid dexamethasone produces both myotube and skeletal muscle atrophy with concurrent elevations in MAFbx and MuRF-1 mRNA [121]. This depends on ligand attachment to the glucocorticoid receiver and upregulation of the transcription factors FOXO3a and FoXO1 for MAFbx and MuRF1, respectively [121]. Treatment with the GC receptor antagonist RU 38486 prevented sepsis-induced upregulation of MAFbx1 and MuRF1 mRNA in EDL muscle [124]. Finally, both atrogin-1 and MuRF1 mRNA increase with inflammation, as observed in the gastrocnemius of a rat model of sepsis, where it was upregulated in a dose- and time-dependent manner, and the increase was prevented by IGF-1 administration, but MuRF-1 was unchanged [125].

### 5.2. Ubiquitin-Proteasome

Muscle protein degradation is governed by a proteolytic strategy consisting of four main mechanisms: the lysosomal proteolytic system, calcium-dependent calpains, caspases, and the ubiquitin–proteasome network. The latter is the primary regulatory mechanism during skeletal muscle atrophy [122].

It has been established that the gene expression patterns of this system are very intense during muscle atrophy. This reaction occurs by increasing the expression of genes involved in conjugating proteins to ubiquitin through the ubiquitin–proteasome system for degradation [92]. The ubiquitin proteolysis system was initially assumed to degrade old, damaged, misfolded, or misassembled proteins, but recently this system has been involved in the monitoring of the accumulation of numerous functionally regulatory proteins, including oncoproteins, transcription factors, cell growth factors, signalling transductors, and cell cycle proteins [67].

This biodegradation through the ubiquitin–proteasome complex occurs in three steps, the first being the covalent binding of ubiquitin polypeptides to the protein to be degraded, then the ubiquitin–ubiquitin coupling to produce ubiquitin polymers and finally the breakdown of the ubiquitin-tagged protein by the 26S proteasome system [67].

After conjugation, the ubiquitin-activating enzyme (E1) activates ubiquitin, allowing the protein to be moved to a ubiquitin transporter protein (E2) [66]. E2 interacts with an ubiquitin ligase (E3) to mediate the transfer of ubiquitin to the protein receptor, signalling the target substrate for proteasomal breakdown [66].

The tight regulation and specificity depend on the function of E3 ubiquitin ligases [88]. These enzymes recognize a specific protein degradation signal, bind to it, and catalyse ubiquitin binding to a lysine residue or the NH2-terminal amino group of the protein [92]. This ubiquitin conjugation to a substrate protein is repeated until a minimum of four ubiquitin molecules is bound to the target protein [122]. The 26S proteasome recognizes this pattern of proteins and ubiquitin as a signal to break down the substrate protein [122,126].

Two muscle-specific ubiquitin ligases, atrogin-1 and MuRF1, are biomarkers of muscle wasting since they are up-regulated in various catabolic derangements [92]. In a study of 20 healthy subjects on 20 days of rest in bed, the thickness of the quadriceps femoris muscle and the cross-sectional area decreased, an accumulation of ubiquitinated proteins was observed, and two ubiquitin ligase genes, Cbl-b and atrogin-1, were upregulated [127].

### 5.3. Calpain and Caspase-3

Caspase-3 and calpain are proteolytic proteins known to degrade contractile proteins [128,129]. Caspase-3 is an enzyme that cleaves proteins at cysteine residues leading to apoptosis [130,131]. Calpains are calcium-dependent non-lysosomal proteases [132].

In sepsis, caspase and calpain participate in diaphragmatic debility [129]. In a rat model of sepsis, diaphragmatic levels of active calpain and caspase-3 were increased [129]. Sepsis-related muscle proteolysis was suppressed by the calpain blockers calpeptin or BN82270 or the NF-κB inhibitor curcumin; nevertheless, this did not decrease MAFbx1 or MuRF1 mRNA [121]. In a rat model of mechanical ventilation, calpain and caspase-3 were activated in the diaphragm and atrophy of various types of fibres was observed [128]. The two effectors are connected because calpain knockdown in the diaphragm prevented caspase-3 activation and reversal [128].

In addition, as previously described, AMPK signalling is an effector of calpains and caspase-3 in C2C12 myotubes [94]. In septic subjects with ICU-acquired muscle debility and atrophy, the calpain systems and levels of apoptosis in the skeletal muscle were altered [132].

## 6. Summary and Future Directions

Inflammation in critically ill patients affects all the pathways of regulation and counter-regulation hormones, from its secretion, signal transduction, and the effectors of muscle breakdown. All the pathways and their catabolic and anabolic effects are integrated in Figure 1 and Figure 2. Insulin, insulin-like growth factor-1, and growth hormone stimulate protein synthesis in muscles, but counter-regulatory hormones counteract these effects. GC and TNF-α, which are secreted during critical illness, promote protein breakdown in muscles, while glucagon does not affect it. In many trials, patients were supplemented with IGF-1 or GH, but it had no positive effect in critically ill patients. However, these trials did not have endpoints centred on muscle mass, muscle strength, or functional status of the patients [45,46,56,57,58,133].

In addition, all signals that translate hormones and their effectors were reviewed. Some experimental trials were conducted, and it was shown that GSK-3B modulates the severity of inflammation and the effect on Atrogin-1 and MuRF1 [101,105]. Furthermore, arginine and leucine can activate mTORC1 recruitment in lysosomes [118]. Finally, it was demonstrated how sepsis and bed rest can affect the activity of the effectors of protein breakdown [127] and how GC receptor antagonist RU 38486 prevented sepsis-induced MAFbx and MuRF1 mRNA upregulation in muscles [121,124].

Most studies have been directed at the amount of proteins delivered and outcomes like mortality [19,20,21]. There is conflicting evidence on the quantity of proteins delivered and the effect on muscle mass and strength [133,134,135,136,137]. Based on the findings reviewed, new trials can be conducted to modulate these pathways and observe the effects on muscle mass and function.

## 7. Conclusions

Several factors affect muscle metabolism despite the amount of proteins needed to maintain protein homeostasis in critically ill patients, whose actual amount still needs to be determined. Many of these cytokines and hormones have been tested through clinical trials in critically ill patients without clear results. Many of these studies are old and have results that can be changed with current knowledge and the ability to measure muscle and lean body mass. New studies with other results are needed to know if these hormones with actual protein and calorie requirements can help prevent or palliate sarcopenia in critically ill patients.

## Figures and Tables

**Table 1:** Main effects of different hormones in muscle proteins.

Hormone	Effects
Insulin	Inhibits gluconeogenic precursor flow to the liver. Stimulates protein synthesis.
Growth Hormone	Increases muscle mass and strength. Increases protein synthesis. Increases amino acid intake.
IGF-1	Integrates anabolic and catabolic responses. Inhibits protein breakdown.
Glucocorticoids	Decreases protein synthesis rate. Increases protein breakdown rate.
Glucagon	No effect.
TNF-alpha	Increases protein catabolism.
Adrenaline	Increases insulin and glucagon liberation. Suppresses protein breakdown.
HIF-1	Increases oxidative metabolism. Increases liberation of cytokines (TNF-alpha).

IGF-1: insulin-like growth factor-1. TNF-alpha: tumour necrosis factor alpha. HIF-1: hypoxia inducible factor-1.

## Data Availability

Not applicable.

## Abbreviations

AMPK	AMP-Activated Protein Kinase
eIF	eukaryotic Initiation Factor
FFM	Fat Free Mass
FM	Fat Mass
GC	Glucocorticoids
COPD	Chronic Obstructive Pulmonary Disease
GH	Growth Hormone
GLP-1	Glucagon Like Factor-1
GLUT-4	Glucose Transporter 4
GSK3B	Glycogen synthase kinase 3 β
HIF	Hypoxia Inducible Factor
IGF-1	Insulin-like Growth Factor
IGFBP3	IGF Binding Protein 3
IL-1	Interleukin 1
LPS	Lipopolysaccharide
MAFbx1	Muscle Atrophy Box 1
mTOR	Mammalian Target of Rapamycin
MuRF1	Muscle RING Finger 1
NF-KB	Nuclear Factor Kappa
PI	Phosphatidylinositol
P70S6k	Ribosomal protein S6 kinase 1
SOFA	Sequential Organ Failure Assessment
TNF	Tumour Necrosis Factor
